# MRI detects coronary vessel wall thickening with age in healthy subjects

**DOI:** 10.1186/1532-429X-13-S1-O47

**Published:** 2011-02-02

**Authors:** Andrew D Scott, Jennifer Keegan, Raad H Mohiaddin, David N Firmin

**Affiliations:** 1 Imperial College London, London, UK; 2 The Royal Brompton and Harefield NHS Foundation Trust, London, UK

## Objective

To investigate the effects of ageing on the coronary vessel wall of healthy subjects using MR.

## Background

Autopsy studies [1,2] and x-ray CT in older subjects with suspected disease [3] have demonstrated increasing coronary vessel thickness with age. There is, however, a need for a radiation-free non-invasive technique for use in longitudinal studies of coronary vessel wall morphology. Recently 3D MR coronary vessel wall imaging with retrospective beat-to-beat respiratory-motion-correction (B2B-RMC)[4], which uses 3D low resolution data acquired immediately before the main imaging data to retrospectively correct respiratory motion, has demonstrated great promise [5]. We propose that 3D high resolution MR with B2B-RMC can demonstrate coronary vessel wall thickening with age in healthy subjects.

## Methods

21 healthy subjects with no history of cardiovascular disease (mean age 39±13, range 22-62, 11 female) were recruited. Studies were performed on a 1.5T Siemens Avanto scanner. Cross-sectional vessel wall imaging was performed in the proximal right coronary artery (<40mm from origin) using a 3D spiral acquisition with B2B-RMC (0.7x0.7x3mm resolution, 8 slices reconstructed to 16x1.5mm, duration 600 cardiac cycles assuming 100% respiratory efficiency) and alternate R-wave cardiac gating. Data were acquired in the coronary rest period, as determined from a cine acquisition in the imaging plane. Circular regions of interest were drawn around the inner and outer coronary vessel wall on one central slice from each 3D acquisition. Average vessel wall thickness and wall/outer wall (lumen + vessel wall) area (W/OW)[6] were calculated.

## Results

**Figure 1:**
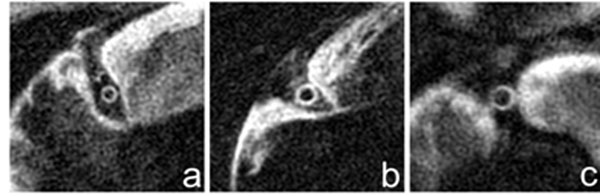
Example images obtained from three male healthy subjects aged 24 (wall thickness 1.02mm, W/OW 0.69) (a), aged 40 (wall thickness 1.38mm, W/OW 0.73) (b) and aged 59 (wall thickness 1.53mm, W/OW 0.70) (c).

**Figure 2:**
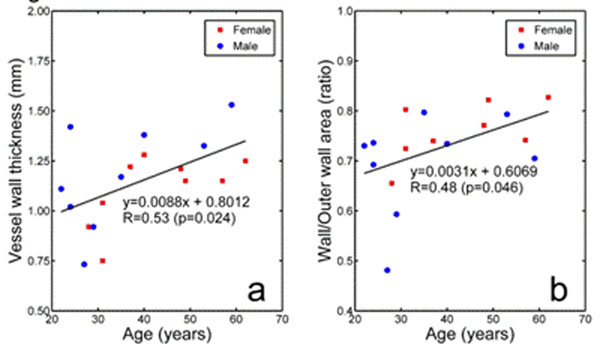
The effects of ageing on the coronary vessel wall as assessed by high resolution MR imaging. Vessel wall thickness (a) and W/OW (b) demonsrate a significant positive correlation with age.

Example images are shown in figure 1. In three subjects (14%) the images were rejected due to poor image quality caused by cardiac or respiratory motion. In the remaining 18 subjects, mean vessel wall thickness was 1.14±0.22mm and mean W/OW was 0.727±0.085. Vessel wall thickness and W/OW increase by 0.088mm (R=0.53,p=0.024,figure 2.a) and 0.031 (R=0.48,p=0.048,figure 2.b) per decade respectively.

## Discussion

For the first time using MR, we have demonstrated significantly increasing vessel wall thickness with age in a small cohort of healthy subjects at 0.088mm per decade. The proportion of vessel that is vessel wall (W/OW) also increases with age at 0.031 per decade (R=0.48). The strength of this correlation is similar to that obtained in larger studies of carotid wall thickening in healthy subjects (R=0.50 males and R=0.46 females [7]).
